# Biofeedback Intervention for Stress, Anxiety, and Depression among Graduate Students in Public Health Nursing

**DOI:** 10.1155/2015/160746

**Published:** 2015-04-14

**Authors:** Paul Ratanasiripong, Orawan Kaewboonchoo, Nop Ratanasiripong, Suda Hanklang, Pornlert Chumchai

**Affiliations:** ^1^Department of Advanced Studies in Education and Counseling, California State University, Long Beach, CA 90840, USA; ^2^Faculty of Public Health, Mahidol University, Bangkok 10400, Thailand; ^3^School of Nursing, California State University, Dominguez Hills, CA 90747, USA

## Abstract

Globally, graduate students have been found to have high prevalence of mental health problems. With increasing severity of mental health problems on university campuses and limited resources for mental health treatment, alternative interventions are needed. This study investigated the use of biofeedback training to help reduce symptoms of stress, anxiety, and depression. A sample of 60 graduate students in public health nursing was randomly assigned to either the biofeedback intervention or the control group. Results indicated that biofeedback intervention was effective in significantly reducing the levels of stress, anxiety, and depression over the 4-week period, while the control group had increases in symptoms of anxiety and depression over the same timeframe. As future leaders in the public health nursing arena, the more psychologically healthy the graduate students in public health nursing are, the better the public health nursing professionals they will be as they go forth to serve the community after graduation.

## 1. Background

With increasing mental health problems among graduate students globally, more efforts are needed to help graduate students manage their levels of stress, anxiety, and depression. Graduate students in public health nursing must not only be competent academically and professionally but also strive to have good mental and psychological health.

There have been only few research studies on the mental health and interventions among graduate students, with limited studies on public health nursing students [1–5]. Australian graduate and undergraduate students from two large university campuses were found to have significantly higher levels of distress than the general population [1]. Taiwanese graduate students were found to have high prevalence rate of fatigue which could lead to other psychological distress [2]. A study among graduate students in public health in Greece found high prevalence of mental health problems that negatively impact their coping and academic performance [3]. Previous studies with university students in Thailand have focused mainly on health issues [6–8] with few researches on mental health issues [9–13]. High prevalence of stress, anxiety, and depressive symptoms was found among Thai university students [9, 10].

As future leaders in the public health nursing arena, it is important for graduate students in public health nursing programs to learn strategies and interventions to help them manage their lives and cope with life stressors. Currently, there has been no study done on the mental health of and intervention for graduate students in public health nursing in Thailand or other developing countries.

As with many developing countries, Thailand has limited counseling resources to help with stress, anxiety, and depressive symptoms, especially on university campuses. In addition, the issue of stigma for seeking help from professional counselors for mental health issues still persists in Thailand. Innovative and culturally appropriate alternative interventions are needed. Biofeedback intervention is one possible alternative. A previous study in Thailand found biofeedback intervention to be effective in reducing anxiety and managing stress among undergraduate nursing students [14]. Biofeedback intervention was also found to be effective in helping other university students in other countries with their mental health issues [15, 16]. No previous biofeedback study has been done among university students regarding depression, even though there is a high cooccurrence rate for anxiety and depression.

Biofeedback has been around since the late 1960s. Biofeedback is a mind-body, self-regulation process for improving performance and health. Through biofeedback equipment, the individual can become aware of his or her physiological function so that he or she can learn to modify thoughts, feelings, or behaviors in order to make positive changes to that physiological function. Biofeedback training has been helpful in reducing symptoms of stress, anxiety, depression, and other health conditions [17]. There are many types of biofeedback training, including electroencephalograph (EEG), electrocardiogram (ECG), electromyography (EMG), electrodermograph (EDG), and heart rate variability (HRV). The equipment used for this study is based on HRV biofeedback. By using HRV biofeedback, the individual gain awareness of the involuntary HRV, learn to breathe slowly and feel positive emotions in order to control the HRV, and ultimately attain reduction in stress, anxiety, and depressive symptoms [14].

## 2. Methods

### 2.1. Design and Participants

This experimental study was conducted with graduate students in the public health nursing program at a major public university in Thailand. Based on a priori power analysis by G∗Power, 60 participants were needed for this study [18]. Using parameters of 0.05 alpha, 0.75 moderate effect size, and 0.8 power, the sample size needed per group for *t*-tests was 29 participants.

All 60 participants have bachelor's degrees and were enrolled in one of the graduate programs in the faculty of public health. Participants' age range was between 21 and 52 (M = 34.05, SD = 7.61). Ninety-seven percent of participants were female and three percent were male. Grade Point Average (GPA) was between 3.00 and 4.00 (M = 3.56, SD = 0.25). Among the participants, 45% were in their first year of their graduate students, 22% were in the second year, 17% were in the third year, 10% were in the fourth year, and 7% were in the fifth year.

### 2.2. Procedures

After the study was approved by the university's Ethics Committee for Human Research, graduate students in the Department of Public Health Nursing were recruited to participate in the study. After informed consent was obtained from the volunteer participants, they completed the preintervention survey packet, including the Perceived Stress Scale, State Anxiety Scale, Center for Epidemiological Study-Depression Scale, and brief demographic questionnaire. Then they were randomly assigned to either the control group or the biofeedback intervention group. Specifically, stratified randomization was used for this study. The strata were male and female. For each set of participants, they were randomly assigned to either the control or biofeedback intervention group.

The authors took into account various potentials for biases and addressed them for this study. For potential selection/sampling bias, the authors made sure that omission bias did not occur by recruiting participants from all the graduate students in public health nursing. The final group of participants was representative of the entire student population in the program. For potential measurement and response biases, participants were reminded to answer the questions in the survey packet honestly and that their answers will not have any negative consequences. Participants were also informed that their participation is voluntary and that they can withdraw from the study at any time. Lastly, no incentives were given to the volunteer participants of this study.

Participants in the control group did not receive any training or equipment to use. Participants in the biofeedback intervention group received one training session by the researchers and were each given a portable biofeedback device to use for 4 weeks. The training session focused on helping participants learn to use the portable biofeedback equipment to help in the management of stress, anxiety, and depression. The first step in the training was helping participants become familiar with the equipment and become aware of their baseline HRV. Then participants were instructed to breathe slowly and to feel positive emotions. By using the portable biofeedback equipment, participants were able to receive immediate visual and auditory feedback on how their breathing and positive emotions impact their HRV. Training was completed when each participant was able to sustain a heart-rhythm pattern associated with positive emotions. Participants were instructed to use the portable biofeedback device 3 times per day for 4 weeks and record their practice times on the log.

At the end of the study, all participants completed the postintervention survey packet, including the Perceived Stress Scale, State Anxiety Scale, and Center for Epidemiological Study-Depression Scale. The data collection process for this study was performed by three of the authors. Both the preintervention survey packet and postintervention survey packet were printed out for the participants to complete. All the participants were asked to meet in one of the classrooms on campus to fill out the pre- and postintervention survey packet. For the postintervention survey packet, three participants were not able to come to campus due to their fieldwork and were e-mailed the survey to complete and e-mail back.

### 2.3. Instruments

Participant's level of perceived stress in the past month was measured by the Perceived Stress Scale [19]. With 10 items on a 5-point Likert scale (0 = never, 4 = very often), higher score on the Perceived Stress Scale indicates higher level of perceived stress. The Perceived Stress Scale has been translated and validated for use in Thailand with several studies focusing on Thai university students [13, 14, 20]. The Cronbach's alpha of the Perceived Stress Scale for the present study was 0.81 for the preintervention and 0.81 for the postintervention.

The level of anxiety was measured by the State Anxiety scale of the State-Trait Anxiety Inventory [21]. With 20 items on a 4-point Likert scale (0 = not at all, 3 = very much so), higher score on the State Anxiety scale indicates higher level of current anxiety. The State-Trait Anxiety Inventory has been used widely in Thailand, including university students; this instrument was found to have good internal consistency and concurrent validity [9, 13, 14]. The Cronbach's alpha of the State Anxiety Scale for the present study was 0.94 for the preintervention and 0.93 for the postintervention.

The level of depression was measured by the Center for Epidemiological Study-Depression Scale [22]. With 20 items on a 4-point Likert scale (0 = rarely or none of the time, 3 = most or all of the time), higher score on the Center for Epidemiological Study-Depression Scale indicates higher level of depression. This instrument has been translated and validated for use with the Thai population, including university student population [9, 11, 12, 23]. The Cronbach's alpha of the Center for Epidemiological Study-Depression Scale for the present study was 0.89 for the preintervention and 0.87 for the postintervention.

## 3. Results

### 3.1. Preliminary Analyses

There were no significant differences in the basic characteristics between the biofeedback and the control groups from the independent sample *t*-tests, chi-square tests, and Fisher's exact tests (see Table 1). Independent sample *t*-tests were also used to compare the pretest results of Perceived Stress Scale, STAI-State Anxiety Scale, and Center for Epidemiological Study-Depression Scale between the biofeedback and the control groups; there were no significant differences between the two groups for these three scales (*p* = 0.10, 0.20, and 0.11, resp.).

### 3.2. Stress

**Figure 1:**
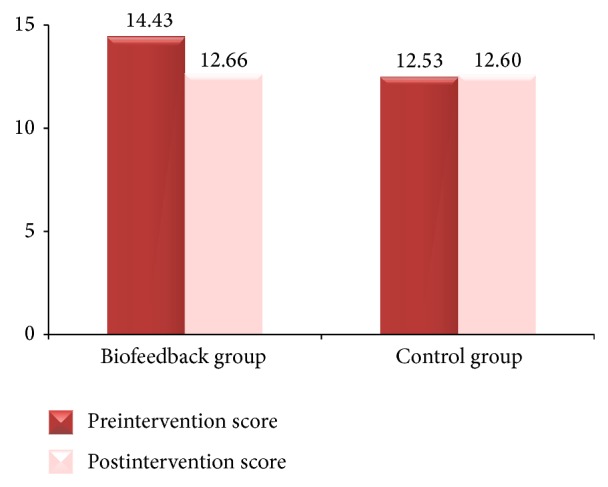
Pre- and postintervention mean scores for Perceived Stress Scale.

In the area of stress, the biofeedback group had a significant decrease in the Perceived Stress Scale over the four-week period, while the control group had a slight increase (see Figure 1). For the biofeedback group, the mean postintervention perceived stress score (M = 12.66, SD = 3.69) was significantly lower than the mean preintervention perceived stress score (M = 14.34, SD = 4.82). For the control group, the mean postintervention perceived stress score (M = 12.60, SD = 5.44) was slight higher than the mean preintervention perceived stress score (M = 12.53, SD = 3.87). A paired-sample *t*-test for the biofeedback group indicated a significant decrease in the perceived stress score: *t*(28) = 2.26, *p* < 0.05, Cohen's *d* = 0.01.

### 3.3. Anxiety

**Figure 2:**
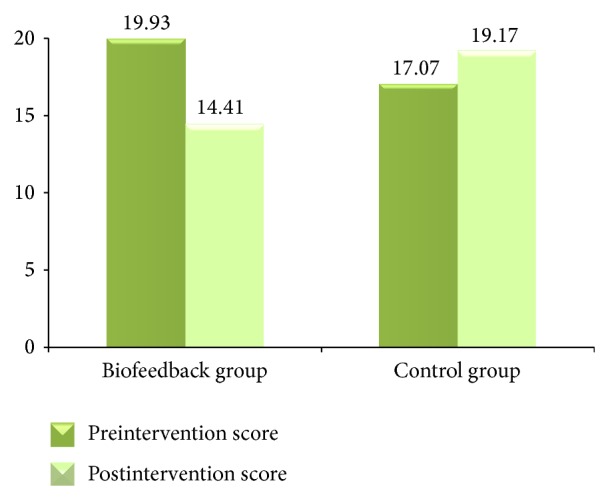
Pre- and postintervention mean scores for State Anxiety Scale.

For anxiety, the biofeedback group had a significant decrease in the STAI-State Anxiety Scale score over the four-week period, while the control group had an increase (see Figure 2). For the biofeedback group, the mean postintervention state anxiety score (M = 14.41, SD = 7.22) was significantly lower than the mean preintervention state anxiety score (M = 19.93, SD = 9.15). For the control group, the mean postintervention state anxiety score (M = 19.17, SD = 9.29) was higher than the mean preintervention state anxiety score (M = 17.07, SD = 7.55). A paired-sample *t*-test for the biofeedback group indicated a significant decrease in the state anxiety score: *t*(28) = 3.47, *p* < 0.01, Cohen's *d* = 0.67. The increase in the state anxiety score for the control group was not statistically significant (*p* = 0.17).

### 3.4. Depression

**Figure 3:**
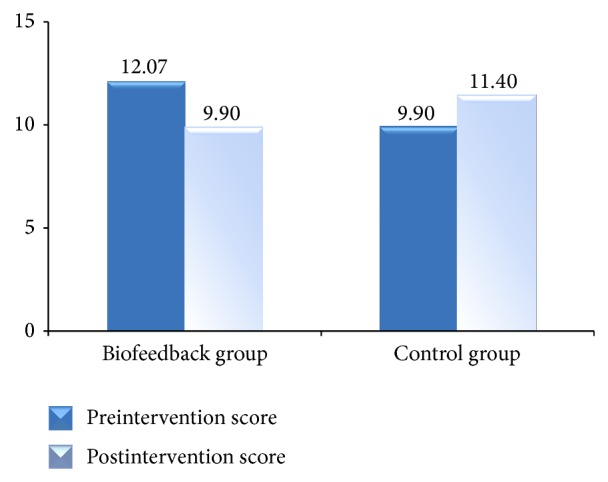
Pre- and postintervention mean scores for CES-Depression Scale.

In terms of depression, the biofeedback group had a significant decrease in the Center for Epidemiological Study-Depression Scale score over the four-week period, while the control group had an increase (see Figure 3). For the biofeedback group, the mean postintervention depression score (M = 9.90, SD = 11.40) was significantly lower than the mean preintervention depression score (M = 12.07, SD = 8.59). For the control group, the mean postintervention depression score (M = 11.40, SD = 7.54) was higher than the mean preintervention depression score (M = 9.90, SD = 7.13). A paired-sample *t*-test for the biofeedback group indicated a significant decrease in the depression score: *t*(28) = 2.90, *p* < 0.01, Cohen's *d* = 0.21. The increase in the depression score for the control group was not statistically significant (*p* = 0.12).

### 3.5. Between Group Comparisons

Multivariate Analysis of Variance (MANOVA) was utilized to determine if there were postintervention differences between the biofeedback and control groups on a linear combination of the three correlated dependent variables—Perceived Stress Scale, STAI-State Anxiety Scale, and Center for Epidemiological Study-Depression Scale. The assumptions of independence of observations and homogeneity of variance and covariance have been met. Bivariate scatterplots were checked for multivariate normality. A significant difference was found, Wilks' lambda = 0.82, *F* (3, 53) = 3.78, *p* = 0.02. Follow-up univariate ANOVAs also confirmed that the STAI-State Anxiety, when examined alone, was significantly different between the two groups, *F* (1, 55) = 4.56, *p* = 0.04. The biofeedback intervention group had significantly lower STAI-State Anxiety score compared to the control group.

## 4. Discussion

Biofeedback training has demonstrated to be an effective form of intervention to help graduate students in public health nursing significantly reduce their levels of stress, anxiety, and depression after 4 weeks. On the other hand, graduate students in the control group had increases in anxiety and depressive symptoms over the same period. The results from this study confirmed previous biofeedback studies on the reduction of stress and anxiety among study participants [14, 16] and also expanded to include the significant impact of biofeedback on depression.

Graduate students in public health nursing face many challenges including demands from academic coursework and research in addition to other life stressors [3]. At worst, unmanageable stress could lead to violent behavior, severe anxiety could lead to incapacitation, and severe depression could lead to suicide. Symptoms of stress, anxiety, and depression among graduate students in public health nursing need to be managed so that they do not negatively impact health, relationships, and academic performance.

In looking at the postintervention differences between the biofeedback group and the control group, results indicated clearly that biofeedback intervention had the most significant impact on the anxiety levels of the participants. However, the postintervention differences between the two groups for stress and depression were not statistically significant; this may be partly due to the duration of the study.

There are some limitations for this study. Even though the participants were randomized into the intervention and control groups, all participants were from one university campus. Additionally, only three percent of the participants were male. Future studies should attempt to recruit more male participants as well as include graduate students in public health nursing from a few university campuses in Thailand and other countries to increase the generalizability. A longitudinal study with annual follow-up with participants could provide further details on the longer-term impact of biofeedback intervention on their mental health.

## 5. Conclusion

In conclusion, academic programs in public health nursing need to be proactive in providing their graduate students with tools and resources to better manage their mental health issues. With increasing severity of mental health problems on university campuses and limited resources for mental health treatment, alternative interventions are needed. Biofeedback intervention is a cost-effective tool to help graduate students in public health nursing manage their stress, anxiety, and depression [24]. As future leaders in the public health nursing arena, the more psychologically healthy the graduate students in public health nursing are, the better the public health nursing professionals they will be as they go forth to serve the community after graduation.

## Figures and Tables

**Table 1:** Basic characteristics of the biofeedback group and the control group.

	Biofeedback	Control	*p* value
	(*n* = 30)	(*n* = 30)	
Age	M = 34.9	M = 33.2	0.37^a^
GPA	3.52	3.60	0.24^a^
Gender			
Female	97%	97%	0.75^b^
Male	3%	3%	
Year in school			
1st	50%	40%	0.053^b^
2nd	30%	13%	
3rd	7%	27%	
4th	13%	7%	
5th	0%	13%	
Family income			
Good	17%	3%	0.06^b^
Moderate	83%	83%	
Poor	0%	13%	
Health problems			
Yes	17%	23%	0.37^b^
No	83%	77%	

M, mean; ^a^by independent *t-*test; ^b^by chi-square test or Fisher's exact test.
